# Sheep Methane Emission on Semiarid Native Pasture—Potential Impacts of Either Zinc Sulfate or Propylene Glycol as Mitigation Strategies

**DOI:** 10.3390/ani10030395

**Published:** 2020-02-28

**Authors:** Hélio Costa, Eloisa Saliba, Marco Bomfim, Ângela Maria Lana, Ana Luiza Borges, Aline Landim, Carlos Mota, Rafael Tonucci, Antonio P. Faciola

**Affiliations:** 1 Department of Animal Science, Vale do Acaraú State University, Sobral 62.040-370, Brazil; alinelandim@yahoo.com.br (A.L.); carlosmikaell@gmail.com (C.M.); 2 Department of Animal Science, Federal University of Minas, Belo Horizonte 30.123-970, Brazil; saliba@ufmg.br (E.S.); lana@vet.ufmg.br (Â.M.L.);; 3 Regional Scientific Development Scholarship of National Scientific and Technological Development Council (Conselho Nacional de Desenvolvimento Cientifico e Tecnológico—CNPq)—Level C (DCR-FUNCAP/CNPq), Brasilia 38706-400, Brazil; 4 Embrapa Goats and Sheep, Estrada Sobral-Groaíras, Km 04, Caixa Postal 145, Sobral 62010-970, Brazil; marco.bomfim@embrapa.br (M.B.); rafael.tonucci@embrapa.br (R.T.); 5 Department of Animal Sciences, University of Florida, Gainesville, FL 32611, USA; afaciola@ufl.edu

**Keywords:** *Caatinga*, CH_4_, rainy season, SF_6_

## Abstract

**Simple Summary:**

Feed availability for small ruminant production in the Brazilian semi-arid region is characterized by the seasonality of forage production over the year. Large variations of methane (CH_4_) production have been reported among forage types and are mainly explained by the rate of fermentation of plant cell contents and the presence of various plant secondary compounds, notably in heterogeneous pasture. The aim of this study was to evaluate the effects of Zinc sulfate and propylene glycol (PG) on CH_4_ emission, nutrient intake, digestibility, and production in sheep grazing on a native *Caatinga* (Brazilian semi-arid savannah) pasture during the rainy season (from March to June 2014). Fifteen mixed Santa Inês sheep were distributed into three treatments (control, Zn, and propylene glycol supplement) in this 112-day study. CH_4_ emission was measured using the SF_6_ tracer gas technique. Across the months of the trial, organic matter (OM) and neutral detergent fiber (NDF) intakes were greater in March, while the greatest emission of CH_4_ (g/day) was observed in May. Total CH_4_ emission (kg) from March to June (112 days of evaluation) was greater in PG. In conclusion, our results indicate that Zn and PG have no beneficial effects in mitigating sheep CH_4_ emission when grazing *Caatinga*-native pasture in the rainy season.

**Abstract:**

The aim of this study was to evaluate the effects of Zinc sulfate and propylene glycol (PG) on methane (CH_4_) emission, nutrient intake, digestibility, and production in sheep grazing on a native *Caatinga* (Brazilian semi-arid savannah) pasture during the rainy season (from March to June 2014). Fifteen mixed Santa Inês sheep, all non-castrated males, with initial body weight of 19.8 ± 1.64 kg, and 4 ± 0.35 months of age, were distributed in a complete randomized design into three treatments: control (CT)—concentrate supplemented at 0.7% of body weight; CT + 300 mg of Zn/day; and CT + 2.5 mL of propylene glycol/kg LW^0.75^/day. Measurements were done in four periods during the rainy season, with 28 days of interval between each measurement. CH_4_ emission was measured using the SF_6_ tracer gas technique. CH_4_ emission per day was greater in PG than in CT and Zn (*p* < 0.05). However, no additive effect was observed on the intakes of organic matter (OM) and neutral detergent fiber (NDF), or on CH_4_ emission expressed as a function of OM and NDF intakes (*p* > 0.05). Across the months of the trial, OM and NDF intakes were greater in March, while the greatest emission of CH_4_ (g/day and g by g/OM intake) was observed in May (*p* < 0.05). Total CH_4_ emission (kg) from March to June (112 days of evaluation) was greater in PG compared with CT and Zn (*p* < 0.05). Zinc and PG had no effect on total CH_4_ emission when it was expressed per unit of body weight gain or carcass production (*p* > 0.05). The results of this study indicate that Zinc sulfate and propylene glycol have no beneficial effects in mitigating sheep CH_4_ emission. The CH_4_ emissions originated from sheep grazing native *Caatinga* pasture change throughout the rainy season due to fluctuations in availability and quality of pasture biomass. Moreover, the inclusion of zinc sulfate or propylene glycol did not improve animal feed intake, nutrient digestibility, and animal performance.

## 1. Introduction

Methane (CH_4_) production through enteric fermentation is a global matter of concern due to its contribution to greenhouse gases (GHG) accumulation in the atmosphere. Enteric fermentation emissions in Brazil increased nearly threefold in the last 40 years [1]. CH_4_ emissions in the livestock sector come from this natural digestive process, where countries from Latin-America contribute with 20.9 Tg CH_4_/yr, representing about 15% to the global enteric fermentation emissions of 104 Tg CH_4_/yr [1,2]. This process also represents a loss of dietary energy by the animal, with 5–9% of the dietary gross energy being lost this way [3,4]. Therefore, reducing CH_4_ emissions can mitigate the impact of the livestock sector on climate change, improve energy utilization, and animal performance.

Reductions in CH_4_ emissions by livestock are associated with improvements in diet quality, e.g., high nutritive pastures and feed supplementation [5]. Effective mitigation strategies should enable a reduced cost of meat production, which is associated to greater energy utilization, in addition to reductions in CH_4_ emissions per unit of product produced [6,7].

Feed availability for small ruminant production in the Brazilian semi-arid region is characterized by the seasonality of forage production over the year. Because the rainfall is concentrated in a short period of the year, January to May [8,9], the availability and quality of forage are compromised during the dry season. Thus, large variations in CH_4_ emission have been reported among forage types and are mainly explained by the rate of nutrient fermentation and the presence of various plant secondary compounds, notably in heterogeneous pastures in semiarid conditions [10].

Several possibilities for reducing emissions of CH_4_, mainly, with grazing animals have been suggested. Out of these, the most used strategy is the inclusion of feed additives with the objective of manipulating the ruminal environment in order to improve nutrient intake, fermentation efficiency, and increase animal production [11]. The supply of nutrients such as proteins and minerals (e.g., zinc) and greater energy intake (e.g., using propylene glycol) can improve nutrient intake and feed utilization, thereby minimizing energy losses [12].

Zn is generally added to diets to ensure that nutritional requirements are met; however, dietary Zn supply for ruminants often exceeds the actual requirements [13]. It has been observed that the inclusion of 100 ppm of Zn affects animal response to protein supplementation and utilization of low-quality forages by affecting ruminal fermentation traits [14]. The supplementation of 20 mg Zn/kg DM as Zn-methionine in a basal diet containing 34 mg Zn/kg DM for lambs significantly improved fiber digestibility and growth rate [15]. In addition, studies have demonstrated that supplementation of organic and inorganic combinations of Zn may enhance performance and improve health of sheep [13]. These reports indicate that ruminants can be fed high-Zn diets without adverse effects [13].

Propylene glycol (PG) can increase ruminal propionate concentration and consequently decrease CH_4_ production in the rumen [16,17], which is energetically favorable to the animal. In vitro studies, with sheep ruminal content, indicate that the main products of PG fermentation were propanol and propionate [18]. A review performed by Nielsen and Ingvartsen [19] reported that propionate is the predominant end product of PG fermentation. PG addition decreased the acetate:propionate ratio in the rumen because part of PG is metabolized to propionate in the rumen. The fermentation of PG in the rumen is further characterized by producing CO_2_ and significant inhibition of CH_4_ production, resulting in less energy loss [18]. Moreover, in a study with lambs, the addition of 4% dietary PG had no difference in feed intake and performance, possibly due to relatively low dietary protein and diet digestibility [20]. The inconsistency in research findings when Zn and PG were fed to sheep justifies the need for studies evaluating their effects on feed intake, nutrient digestibility, and CH_4_ emissions.

Therefore, our hypothesis was that zinc and propylene glycol would reduce methanogenesis in sheep grazing native pastures in the semiarid region of *Caatinga*. The aim of this study was to evaluate the effects of zinc sulfate and propylene glycol on CH_4_ emissions, nutrient intake, and production from sheep grazing in a native *Caatinga* (Brazilian semi-arid savannah) pasture during the rainy season.

## 2. Materials and Methods

### 2.1. Animal Use

Animal handling and procedures involving all experimental animals were undertaken according to protocols approved by the Ethics Committee on Animal use of the Federal University of Minas Gerais (CEUA/UFMG, no. 321/2013).

### 2.2. Characterization of the Experimental Area

**Figure 1:**
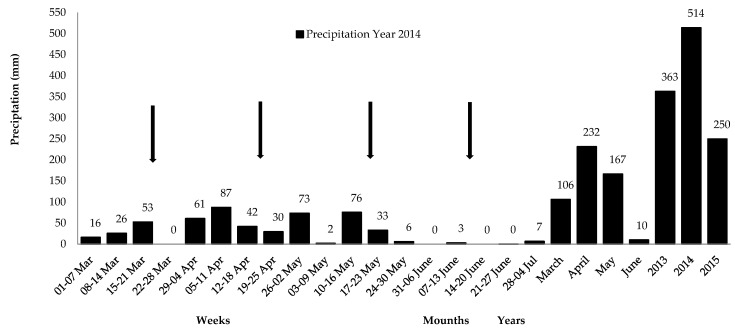
Weekly and monthly precipitation from March to June 2014 and yearly precipitation during the assessment in 2013, 2014, and 2015. Arrows indicate collection weeks in each month. Source: [22].

The study was conducted at Embrapa Goats and Sheep, located in the state of Ceará, Northeast Brazil (3°45’51.59” S and 40°21’04.24” W, 92 m asl). Eight hectares of a native *Caatinga* pasture area were thinned according to Araújo Filho [21]. The area’s predominant soils were litolic dystrophic, planosol and non-calcium brown. The experiments were executed during the rainy season (March to June 2014), with a precipitation of 514 mm (Figure 1) and average temperature and air humidity of 26.5 °C and 78.0%, respectively [22].

### 2.3. Animals and Experimental Treatments

Fifteen mixed Santa Inês sheep, all non-castrated males, with initial body weight of 19.8 ± 1.64 kg, and 4 ± 0.35 months of age, were distributed in a complete randomized design into three treatments: control (CT)—concentrate supplemented at 0.7% of body weight; CT + 300 mg of Zn/day; and CT + 2.5 mL of propylene glycol/kg LW^0.75^/day.

Animals were kept in continuous stocking and weighed weekly to monitor daily body weight gain and supplementation feeding. All animals had free access to mineral salt, with compositions of: Ca = 82.0 g/kg, Co = 30.0 mg/kg, Cu = 350 mg/kg, Cr = 11.7 mg/kg, S = 11.7 g/kg, P = 60.0 g/kg, I = 50.0 mg/kg, Mn = 1200 mg/kg, Mo = 180 mg/kg, Se = 15 mg/kg, Na = 132 g/kg, and Zn = 2600 mg/kg.

The daily Zn dosage required to increase concentration in ruminal fluid at 300 mg Zn/day was calculated. The amount of Zn was established considering the concentration in the mineral salt and the addition of ZnSO_4_.7H_2_O. The amounts of salt and zinc sulfate supplied to the animals were weighed and mixed prior to being provided and adjusted to not contain leftovers. For Zn supplementation, the procedures described by Arelovich et al. [23] and the maximum tolerable level of toxicity for sheep according to NRC (2007) [24] were used. PG was supplied at 2.5 mL × kgLW^0.75^ animal/day [17], and mixed directly into concentrate. PG supply was adjusted weekly, according to the group’s average body weight in kgLW^0.75^ (*n* = 5).

A stocking rate of 0.4 ha/head was used, considering an animal of 30 kg of LW [21]. The animals were taken to the pasture at 07:00 and brought back at 16:00, when they were supplemented according to treatments. The concentrate was composed of corn (540 g/kg DM), soybean meal (451 g/kg DM) and limestone (9.0 g/kg DM), formulated as recommended by the NRC (2007) [24], for finishing lamb with a predicted average daily gain of 150 g.

### 2.4. Forage Availability and Botanical Composition

Before the start of the experimental period, the occurrence of the main forage groups and species were determined using the method proposed by Araújo Filho [21], with a frame measuring 0.25 m^2^ and systematically arranged along lines, every 4 m, totaling 50 sampling points. The percentage of the main forage species of the herbaceous stratum in the area was analyzed. Forage availability in weight was estimated by collecting the forage from the herbaceous stratum contained within the frame every 12 m. The material was weighed and oven dried at 55 °C for 72 h to calculate DM/ha availability (Table 1).

### 2.5. Determination of Nutrient Intake and Pasture Sampling

Four intake and digestibility measurements were carried out in the rainy season during the months of March to June, with an interval of 28 days between periods. To determine total intake, external indicator LIPE^®^ (patent No BR0304736-9) was administered orally in the morning, at a dose of 0.25 g per animal/day, for a period of 7 days, with 2 days for adaptation and stabilization of the indicator in the gastrointestinal tract, and 5 days for fecal collection [25]. Fecal samples were collected directly from the animals’ rectum, stored in plastic bags and frozen in a freezer at −20 °C. Samples composed by animal, by period, were dried at 55.0 °C for 72 h and milled so as to determine LIPE^®^ concentration in the feces and estimate fecal production (FP), as per the equation below:

Fecal production, g/day = (LIPE^®^ supplied, g/LIPE^®^ recovered in feces, g) × fecal DM, g/kg (1)

To assess digestibility and forage chemical composition (Table 2), two ruminally-cannulated adult sheep were used, with mean body weight of 34.5 ± 2.1 kg. Ruminal extrusa samples were collected as described by Olson [26], for 5 consecutive days in each experimental period, starting 1 day before feces collection using the animals used for intake determination. The collection procedure consisted of emptying all ruminal content, which was stored in clean plastic containers. Then, animals were allowed to graze for 1 hour. After this time, all ruminal extrusa were collected. After that, the ruminal content initially removed was returned to the rumen.

Total OM intake was calculated using fecal DM production estimated by a LIPE^®^ indicator by [28]:

Intake (g OM/day) = Production of fecal DM (g/day)/1−IVOMD/100(2)

### 2.6. Determination of Enteric CH_4_ Emission

Gases were collected in four occasions, with a 28-day interval, right after the intake measurements. CH_4_ emitted by the animals was determined by the SF_6_ tracer gas technique [29] with adjustments for measurements in sheep. The SF_6_ permeation tubes that were used had average permeation rates of 1099 mg/day. The tubes were deployed in the animals’ reticulum (per dosing), 28 days before the first collection. The collection was performed for two consecutive 48 h periods, per animal, monthly. The SF_6_ capsules were calibrated to release 1–2 mg of SF_6_ every 24 h, considering that the SF_6_ and CH_4_ followed similar emission patterns. Quantified gases were emitted from the mouth and nostrils of the animal.

**Figure 2:**
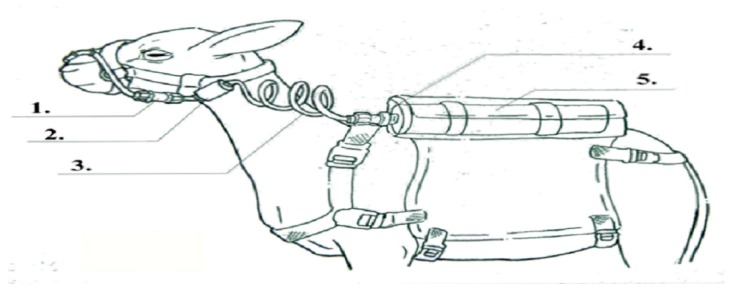
Components of the device used for measuring CH_4_ emission. Illustration of components to collect CH_4_: **1.** Flow control valve; **2.** particle filter; **3.** spiral hose; **4.** quick connect; **5.** stainless cylinder.

To avoid alterations in normal feed-behavioral, sheep were previously acclimated to the devices used for measuring CH_4_. The devices were equipped with a nylon halter with three fixation points (mouth, lower jaw, and neck root behind the ears), a flow control valve, a particle filter, and a spiral hose with a quick connect in one end and a 445-cm^3^ stainless cylinder (Figure 2). The other end of the hose, responsible for capturing the gases emitted to be stored in the cylinders, was fixed on the halter in a leather flap attached to the halter and placed over the muzzle near the animal’s nostrils and mouth.

The cylinder was coupled to a bag attached to the back of the animal. The cylinder was cleaned previously with pure nitrogen 5.0 (degree of purity: 99.9%, for application in chromatography with a Flame Ionization Detector—FID) and vacuum-emptied to contain negative pressure prior to each sample collection. The flow regulators were calibrated to allow a remaining vacuum in the cylinder of about 500 mb (which represents half of the total cylinder volume) at the end of the sample collection period. The inlet regulator was calibrated for each collection period of 4 consecutive days. Two cylinders (blanks) were distributed in the area at a height similar to that of the grazing animals’ reach for correction of the gases contained in the environment. The flow of CH_4_ emitted by the animal was calculated by correlating it with the SF_6_ flow, since the tracer gas release rate in the rumen had been determined previously [29].

CH_4_ emission values were calculated in g/day. Based on these results, inter-relationships were made with productive parameters, and CH_4_ emission was determined as a function of OM and NDF intakes (g/day and g/kgLW^0.75^), total CH_4_ emission as a function of body weight gain in the period (WGP), and per kilogram of cold carcass.

### 2.7. Chemical Analyses

Ruminal extrusa and fecal samples were dried at 55 °C for 72 h, and, together with the concentrate, milled in a knife mill with 1 mm sieves. They were analyzed for DM (method 934.01), ash (method: 938.08), CP (method 968.06) in Nitrogen analyzer (Leco^®^ CN628, St Josesh, MI, USA), and ether extract (EE) (method 920.39) according to the Association of Official Analytical Chemists International (AOAC 1990) [30]. OM was calculated as the difference between DM and ash content, Ash-free values of Neutral Detergent Fiber (aNDFom-NDF) and ADF were analyzed according to Goering [31], with adaptation for autoclave analysis according to Senger [32]. Acid detergent lignin (ADL) content was analyzed (method 973.18D) according to AOAC (1990) [30], neutral detergent insoluble nitrogen according to Licitra [33], and KL was analyzed by acid hydrolysis [34]. Total tannins were analyzed using the Folin–Ciocalteu method [35]. Concentrations of CH_4_ (ppm) and SF_6_ (ppt) were obtained by gas chromatography using electron capture (350 ºC) and flame ionization (250 °C) detectors, respectively [36].

### 2.8. Statistical Analyses

A completely randomized design, with five replications (animals) per treatment, was used for the evaluation of CH_4_ emission in g/day and as a function of OM and NDF intakes (g/day and g/kgLW^0.75^). Statistical differences of treatment parameters and periods were determined by the model below:

Y_ijkl_ = μ + T_i_ + a_ij_ + P_k_ + (T*P)_ik_ + e_ijkl_

where µ = overall mean; T_i_ = fixed effect of treatments (two degrees of freedom—DF) (i = CT, Zn, PG); a_ij_ = random residual effect associated with animal; P_k_ = fixed effect of period (three DF) (k = March, April, May, June); (T*P)_ik_ = treatment*period interaction (six DF); and e_ijkl_ = experimental error associated with the animal observation in each month.

The following statistical model was used to evaluate CH_4_ emissions as a function of production parameters:

Y_ijk_ = μ + T_i_ + a_ij_ + e_ijk_

where µ = overall mean; T_i_ = fixed effect of treatments (i = CT, Zn, PG); a_ij_ = random residual effect associated with animal; and e_ijk_ = experimental error associated with the observation.

Means were compared by the Tukey–Kramer test, with a significance of 0.05. The Proc GLM procedure of the Statistical Analysis System 9.0 (SAS Inst. Inc., Cary, SC, USA) was used.

## 3. Results

A greater amount of g CH_4_/day was emitted from PG compared with CT and Zn (*p* < 0.05; Table 3). However, no effects (*p* > 0.05) of treatments were observed in OM and NDF intakes or emission expressed in mg CH_4_ relative to OM and NDF intakes (g/day and g/kg LW^0.75^). Among periods, greater OM and NDF intakes were observed in March and greater g CH_4_/day emission was observed in May; the same pattern was observed for CH_4_ in mg/OM intake (g/day and g/kg LW^0.75^; *p* < 0.05). There was no effect of period in CH_4_ emission as a function of NDF intake (*p* > 0.05).

There was no effect of treatments in production parameters (*p* > 0.05; Table 4). For total CH_4_ emission in the period from March to June, totaling 112 days of evaluation, greater values were observed in PG compared with CT and Zn (*p* < 0.05). There was no effect of treatments in kg CH_4_/kg of body weight gain in the period, or kg CH_4_/kg NDF intake (*p* > 0.05).

## 4. Discussion

To our knowledge, studies measuring the emission of CH_4_ in vivo in sheep in conditions of tropical semiarid regions, notably in the *Caatinga* biome, are scarce. It was important to understand how the great diversity of forages may contribute or not to emission of CH_4_, especially in the rainy season, a time of greater abundance of species. Allied to this, a few studies using Zn or PG as modulators in the rumen to mitigate the emission of CH_4_ in vivo in sheep have been reported. We tested the strategic use of these as additives in sheep grazing in areas of heterogeneous pasture aiming to improve the use of pastures and mitigate CH_4_ emission.

The lower OM and NDF intakes starting from April were affected by the quantity and quality of the pasture, which had lower in vitro DM and OM digestibility (Table 2). The maturation of the native *Caatinga* pasture during the rainy season greatly modifies the chemical–physical structure of the feeding environment [37]. According to these authors, there is a decrease in the herbaceous layer, and, at the same time, annual shrubby species grow during the months of January to May; from then on, these species become highly lignified, affecting diet quality.

Holter and Young [38] reported the relationship between CH_4_ emission and several dietary factors such as the diet chemical composition, nutrient intake, and digestibility. The decreased pasture quality (April to June) and lower intake may have contributed to the lower CH_4_ emission, especially in June (Table 3). On the other hand, greater intakes promote a reduction in CH_4_ emission per unit of feed ingested, which is directly related to alterations in the fermentation pathways and/or reduction of retention time [39]. In June, this last aspect seemingly contributed to the lower CH_4_ emission. The data from this study fall within normal ranges. Such an aspect reinforces the notion that differences in CH_4_ yield were related to variations in intake during the months. Another aspect that contributes to lower CH_4_ emission indirectly as a mitigator is the secondary’s compounds [40]. Greater content of total tannins was verified in the pasture component obtained in June. This condition is also closely linked to lower CH_4_ emissions in the period. Changes in intake between low-quality and high-quality forages also resulted in changes in CH_4_ per kg OM intake [41]. According to the authors, the intake level and ruminal outflow, which are often positively correlated, partly explain this variability.

Minerals play an important role in the ruminal environment, e.g., by changing the osmotic pressure, buffer capacity, and dilution rate in the rumen. Kurihara et al. (1997) demonstrated that supplementation with ZnSO_4_ in ruminants’ diets at levels greater than 1000 ppm promoted reductions in ruminal protozoa population, which may result in decreasing CH_4_ emission. Moreover, later studies showed that greater levels of Zn in the diet increase propionate concentration and reduce the acetate:propionate ratio [14,23].

In this regard, we speculate that Zn addition could have increased propionate concentration in the rumen, which is considered an important hydrogen-competing pathway [12]. However, CH_4_ emissions originating from sheep in the Zn treatment were similar to that of animals receiving CT, with no effects of Zn on CH_4_ mitigation. Both of these treatments emitted lower CH_4_ levels than PG.

In a study investigating the effect of ZnSO_4_ supplementation in diets containing different levels of protein (6.5 and 8.5% CP, and 8.5% CP + 35 g ZnSO_4_/animal/day) for dairy cows, researchers observed that the possible ruminal protozoa reduction caused by zinc sulfate decreased CH_4_ in L/kg DM by 60% [39]. In an experiment evaluating the effects of organic zinc supplementation (ZnSO_4_ and Zn-peptides) in sheep, the authors observed that supplementation with Zn-peptides might result in a greater concentration of metabolizable energy and greater production of short-chain fatty acids [42]. Propylene glycol is metabolized in the rumen to lactate and propionate [43], allowing the capture of oxygen and reduction in CH_4_ production. However, CH_4_ emissions originated from animals on PG were 18.2% greater, which was not expected. By contrast, the greater intake obtained in May contributed to greater emissions of CH_4_ as compared with June, probably due to the consumption of low-quality fractions, which implies longer retention of the fibrous fractions in the rumen.

Overall, tropical forages have greater proportions of fiber compared with temperate species, which contributes to acetic fermentation and greater production of CH_4_ g/day. On the other hand, this type of fiber has low digestibility, as observed in this study (Table 2), when slower fermentation rates were observed, implying lower amounts of substrate for methanogenic microorganisms [44].

The average emission flow in the present study was 16.9 g CH_4_ animal/day, considering that animals had 24.0 ± 1.81 kg LW, an average OM intake of 540 g/day, and 37.8% pasture IVOMD (Table 2 and Table 3), yielding a supply of 204 g digestible OM/day. CH_4_ emissions as a function of OM and NDF intakes (31.3 and 72.7 mg CH_4_/day, respectively) were not affected by the treatments, and the different months in the rainy season, except for the month of May, in which the greatest CH_4_ emission was recorded. Emission rates were greater than the 11.8 g CH_4_ animal/day observed by Leuning et al [45]. Also, in their study, emissions were originated from sheep with 27.0 kg LW, a DM intake of 508 g/animal/day, and pasture DM digestibility of 69.5%.

Evaluating the effect of supplementation with tropical tanniferous legumes as a strategy to mitigate CH_4_ emission in sheep with an average LW of 27.9 ± 2.85 kg, emissions were obtained in the range from 7.80 to 11.3 g CH_4_/day [46]. Another study determined enteric flow of CH_4_ of Somalis sheep with an average LW of 26.8 ± 2.90 kg, and 14.9 and 11.4 g CH_4_ animal/day were observed for animals in enriched or unenriched thinned *Caatinga* areas, respectively [47]. Also, in that study, a flow of 13.0 mg CH_4_/g OM consumed by animals in an enriched thinned *Caatinga* area during the rainy season was observed. Therefore, improvements in pasture use efficiency and of supplementation strategies may reduce CH_4_ emissions that originated from sheep.

Total CH_4_ emissions were 22.3% greater for PG treatment. These greater emissions were not expected, as previously discussed, because the inclusion of PG in the concentrate might have changed the fermentation pattern, resulting in lower CH_4_ emission. We believe that propylene glycol could have escaped ruminal fermentation and absorbed in the small intestine and converted to glucose in the liver [43]. Previous studies have indicated that propylene glycol can be rapidly absorbed from the rumen without affecting ruminal fermentation [20,48]. This aspect could have occurred, and although VFA was not evaluated, probably there were no changes in the acetate:propionate ratio, since acetate is directly related to CH_4_ production. Average CH_4_ emission per kg of cold carcass, or kg of product, was 0.243 kg. The concept of CH_4_ emission intensity, based on emissions per unit of product, seems to reflect more precisely the effects of mitigation practices in intake, CH_4_ emission, and productivity of an animal [7].

The animals in this study had unsatisfactory performance, with lower body weight gain and carcass yields than expected. However, CH_4_ emissions based on productive parameters were not affected by the addition of ZnSO_4_ and propylene glycol. It is important to correlate CH_4_ emissions with productive parameters, as some studies indicate that ruminants raised on native pastures are the greatest CH_4_ emitters. For the development of inventories and establishment of mitigation practices, this information should be associated with the carbon footprint for production of meat and other animal products.

## 5. Conclusions

The results of this study indicate that Zinc sulfate and propylene glycol have no beneficial effects in mitigating CH_4_ emissions from sheep grazing native pastures in the *Caatinga* region. CH_4_ emissions from sheep grazing native pastures in the *Caatinga* region change throughout the rainy season due to fluctuations in availability and quality of pasture biomass. Moreover, the inclusion of zinc sulfate or propylene glycol did not improve animal feed intake, nutrient digestibility, and animal performance.

## Figures and Tables

**Table 1:** Herbaceous stratum availability, expressed as DM (Dry Matter), and floristic composition of *Caatinga*-native pasture in the rainy season.

DM Availability, kg/ha			Floristic Composition, g/kg	
Legumes	Grasses	Total	Legumes	Grasses
1364	533	1897	719	281

**Table 2:** Composition of ruminal extrusa samples^†^ collected from sheep on *Caatinga*-native pasture during the rainy season.

Variables	Periods				Concentrate ^β^
	March	April	May	June	
Dry Matter ^¥^, g/kg	118	128	142	158	877
g/kg DM					
OM	819	810	798	819	913
Crude Protein	192	187	176	131	254
Neutral Detergent Insoluble Nitrogen (NDIN)	2.99	2.87	3.03	3.01	3.04
NDIN, % of total nitrogen	98.3	96.3	108	145	74.6
Ether Extract	76.0	76.5	86.8	111	64.0
Neutral Detergent Fiber	524	590	610	564	159
Ash-free values of Neutral Detergent Fiber (aNDFom-NDF) ^‡^	437	496	504	478	113
Acid Detergent Fiber	430	476	487	473	103
Hemicellulose	94.1	114	123	91.5	56.1
Cellulose	208	250	261	243	45.6
Acid Detergent Lignin	35.4	45.4	52.5	37.8	11.3
Klason Lignin	40.7	50.4	65.4	52.8	17.8
Total tannins	0.64	8.14	8.33	14.8	-
In Vitro Dry Matter Digestibility ^†^	537	408	424	441	954
In Vitro Organic Matter Digestibility	468	333	353	359	939

^†^ Ruminal extrusa samples collected prior to rumen emptying after a 1-hour grazing in thinning *Caatinga* area, ^†^ In Vitro Dry Matter Digestibility to according to [27]; ^β^ Corn, soybean meal, and limestone; ^¥^ Dry matter on a as fed basis; ^‡^ aNDFom-NDF assayed with a heat stable amylase and expressed exclusive of residual ash NDF.

**Table 3:** Effect of zinc or propylene glycol supplementation on nutrient intake and CH_4_ emission in sheep on *Caatinga*-native pasture.

Variable ^£^	Treatment ^‡^			Period ^β^				SEM ^¥^	P-value ^†^		
	CT	Zn	PG	Mar	Apr	May	Jun		T	P	T × P
Intake, g/day											
OM	527	542	551	607 ^a^	539 ^b^	509 ^b^	505 ^b^	9.39	0.56	<0.01	0.57
NDF	231	236	246	279 ^a^	233 ^b^	235 ^b^	204 ^c^	5.47	0.33	<0.01	0.52
CH_4_ emission											
g/day	15.8 ^b^	15.6 ^b^	19.2 ^a^	16.5 ^ab^	17.2 ^ab^	18.8 ^a^	15.0 ^b^	0.56	0.01	0.04	0.11
mg/OM	30.0	29.0	35.0	27.9^b^	32.2 ^b^	37.3 ^a^	29.7 ^b^	1.24	0.09	0.04	0.11
g/OMkgLW^0.75^	0.29	0.30	0.35	0.27^b^	0.31 ^b^	0.37 ^a^	0.31 ^b^	0.01	0.09	0.04	0.12
mg/NDF	69.9	68.9	79.4	61.1	75.6	81.0	73.3	2.89	0.17	0.07	0.18
g/NDFkgLW^0.75^	0.68	0.71	0.79	0.60	0.73	0.80	0.77	0.03	0.25	0.06	0.17

^a, b^ Means in the same row followed by different letters are different by the *Tukey–Kramer* test (*p* < 0.05). ^‡^ CT = control; Zn = ZnSO_4_.7H_2_O addition for supply of 300 mg Zn/day in the salt; PG = addition of 2.5 mL/g/kgLW^0.75^ /animal/day of propylene glycol mixed with the concentrate. ^β^ Mar = March Apr = April; May = May; Jun = June. ^£^ OM = organic matter; NDF = neutral detergent fiber. ^¥^ SEM = standard error of the mean; ^†^ T = treatments; P = period; T × P = interaction between treatments and periods.

**Table 4:** Effect of zinc or propylene glycol supplementation on CH_4_ emission from sheep in sheep on *Caatinga*-native pasture and in relation with production parameters.

Variable	Treatment ^‡^			SEM ^¥^	*P*-value
	CT	Zn	PG		
	Production parameter				
Cold carcass, in kg	7.45	8.16	8.33	0.270	0.37
Body weight gain in the period, in kg	3.33	4.08	4.31	0.365	0.29
	CH_4_ relation with production parameters				
^β^ Total CH_4_, kg	1.81 ^b^	1.71 ^b^	2.20 ^a^	0.057	0.01
kg CH_4_/kg of total body weight gain	0.688	0.411	0.700	0.072	0.09
kg CH_4_/kg of cold carcass weight	0.248	0.209	0.271	0.009	0.12

^a, b^ Means in the same row followed by different letters are different by the *Tukey–Kramer* test (*p* < 0.05). ^‡^ CT = control; Zn = ZnSO_4_.7H_2_O addition for supply of 300 mg Zn/day in the salt; PG = addition of 2.5 mL/g/kgLW^0.75^ /animal/day of propylene glycol mixed with the concentrate. ^β^ Total CH_4_ = total CH_4_ emission, in kg, during the rainy season (March-June, for 122 days). ^¥^ SEM = standard error of the mean.
